# On the Ethereal Solution of Gun Cotton

**Published:** 1848-09

**Authors:** 


*On the Ethereal Solution of Gun Cotton.* By Edward Parrish and W. W. D. Livermore.

We select the portion of an article bearing the above heading, which relates to the mode of preparing the ethereal solution of gun cotton for surgical purposes.—Ed. Buff. Med. Jour.

"The discussion, as to priority of discovery, has been continued in several subsequent numbers of the same Journal.* On the merits of this discovery, we have nothing to say, nor do the numerous uses of this solution in surgical practice fall within the sphere of our investigation. What particularly concerns us as pharmaceutists is its mode of preparation, and upon this subject, both the writers referred to have left us in the dark. As soon as a demand was created for the article, Dr. Maynard's formula for preparing it was placed in the hands of Maynard & Noyes, Druggists, Boston, who commenced the manufacture of it on a large scale, and measures were taken to introduce it in this city and elsewhere. As it became extensively known and esteemed among physicians and surgeons, of course a number of chemists attempted its preparation. This has been attended with varying success from ignorance of the precautions necessary to be observed, and from the absence of correct formula.

The following observations are the result of a series of experiments in making the solution which have several times disappointed us: as far as they go, they are freely offered for the benefit of others who may be disposed to attempt it.

1st. Ordinary commercial gnn cotton is not soluble in ether.

2nd. The best formula that we have tried for the preparation of this solution, is as follows:

Take of Nitric acid, sp. gr. 1,452,
Sulphuric acid, (Commercial) each, 1 fluid ounce,
Cleansed and bleached cotton, 2 drams.

Thoroughly saturate the cotton with the acids, and macerate for twelve hours. Then wash the cotton, dry it rapidly by artificial heat, in the shade, and dissolve it in

Sulphuric ether, one and a half pints.

---

*The Boston Med. & Surg. Journal.

3d. Gun cotton as thus prepared, will lose its solubility entirely, by being kept a few days, or particularly by being exposed to the sun's rays.

4th. The gun cotton prepared, as above, is entirely soluble in the officinal sulphuric ether, though not in the hydrate ether or letheon.

5th. As many groundless objections to the solution are due to its being carelessly or improperly applied, care should be taken to saturate the fabric used in making the plaster with the liquid, and allow it to dry while in close contact with the skin, and where a permanent plaster is required, it is well to apply it over the exterior surface with a brush. When thus applied, a piece of muslin one inch in breadth, and applied over a surface of an inch and a half in length, will sustain a weight of ten pounds, its adhesion not being affected by moisture or temperature.

6th. Some solutions of cotton, though resembling the true *collodion* in appearance, are found to produce a plaster of inferior adhesive power, and which ceases to adhere on being moistened. Such specimens yield a white precipitate upon drying, which appears to be due to the presence of water. The residue, after the evaporation of the best specimens, is nearly transparent in thin sheets, having somewhat the appearance of tissue paper, and is not readily inflammable.—*American Journal of Pharmacy.*

